# Identification of bacteriophage DNA in human umbilical cord blood

**DOI:** 10.1172/jci.insight.183123

**Published:** 2025-07-08

**Authors:** Jennifer A. Sequoia, Naomi L. Haddock, Paw Mar Gay, Layla J. Barkal, Purnima Narasimhan, Nadine Martinez, Virginia D. Winn, Paul L. Bollyky

**Affiliations:** 1 Division of Neonatal and Developmental Medicine, Department of Pediatrics,; 2 Division of Infectious Diseases and Geographic Medicine, Department of Medicine,; 3 Division of Pulmonary, Allergy, and Critical Care Medicine, and; 4 Division of Reproductive, Stem Cell and Perinatal Biology, Department of Obstetrics and Gynecology, Stanford University School of Medicine, Stanford, California, USA.

**Keywords:** Infectious disease, Microbiology, Bacterial infections

## Abstract

Bacteriophages, viruses that parasitize bacteria, are abundant in the human microbiome and may influence human health, in part, through their interactions with bacterial hosts. Whether endogenous bacteriophages or their products are vertically transmitted from mother to fetus during human pregnancy is not known. Here, we searched for bacteriophage sequences from five bacteriophage databases (474,031 total sequences) in cell-free DNA (cfDNA) of paired maternal and umbilical cord blood samples from two independent cohorts. First, we sequenced cfDNA from 10 pairs of maternal and cord blood samples, including four pairs affected by preeclampsia. We validated our findings in a previously published dataset of 62 paired maternal and cord blood samples, including 43 pairs from preterm or chorioamnionitis-affected deliveries. We identified 94 and 596 bacteriophage sequences in maternal and cord blood cfDNA samples from the first and second cohort, respectively. We identified 58 phage sequences across maternal-infant dyads and 581 phage sequences that were unique to a single sample. We did not identify any phage sequences consistently associated with preeclampsia, preterm, or chorioamnionitis-affected samples. This study demonstrated the presence of bacteriophage DNA in human cord blood at birth, providing evidence that the human fetus is exposed to bacteriophage DNA in utero.

## Abstract 2

Shared bacteriophages sequences are present in paired maternal and fetal cord blood samples, suggesting perinatal transmission of these viruses.

## Introduction

Early life microbial exposures are critical for immune education, intestinal development, and the establishment of a healthy microbiome (1–3). While extensive research has been directed toward understanding how neonates acquire their bacterial communities, there is growing interest in the developing virome in early life (4–12).

Bacteriophages (phages), viruses that infect bacteria, are highly abundant in the human body (13, 14). They influence human health and disease by their interactions with microbial communities and through direct and indirect effects on the human immune system (13, 15–18). However, the timing of when neonates are first exposed to bacteriophages or their products is not well defined.

In humans, there are indications that bacteriophages are passed from mother to neonate perinatally (12, 19, 20). Multiple studies have demonstrated phages and phage DNA are present in meconium shortly after birth (7, 8, 19), indicating prenatal transmission as meconium is formed in utero; however, it is difficult to rule out intrapartum or postnatal transmission, as these samples are collected hours to days after delivery (21). Indeed, samples of meconium taken at the time of cesearean section did not reveal a microbial signal (21). Postnatal transmission has been described for bifidophages, which are thought to pass as prophages via bifidobacterial hosts from mother to infant (20). During pregnancy, bacteriophage DNA circulates in maternal blood and thus comes in direct contact with placental tissues, suggesting a potential avenue for prenatal transmission (22).

The prenatal transmission of bacteriophages has been demonstrated in multiple animal models (23–29). The first animal study to indicate vertical phage transmission was from 1928, wherein guinea pigs were injected in the peritoneum or subcutaneous tissue with three different phages, which were subsequently recovered in the heart blood, intestinal contents, or placenta of the pups (23). Subsequent studies have demonstrated phage transmission in mice (27–29), rabbits (24), and rats (25) and have investigated intrauterine (25), intracardiac (25), and intravenous (24–29) routes of administration. These animal studies have generally required high phage titers (11 ×10^9^ plaque forming units per milliliter) and have yielded inefficient transmission (approximately 0.0001%), which may, in part, be attributed to a robust maternal antibody–mediated immune response to the phage (30). While these experimental animal models have demonstrated the potential for maternal-fetal transmission of phage, it is not clear if this mode of transmission is applicable to endogenous phage or whether prenatal transmission of bacteriophages or phage DNA occurs in humans.

Compared with meconium, umbilical cord blood collected at birth can more accurately reveal in utero microbial exposures. Historically, however, it has been difficult to detect phages in blood samples by standard culture protocols, as blood is not a dense site of bacteriophages, and relevant host bacteria are typically not known a priori (31–35).

One alternative approach to study the human phageome is metagenomic sequencing of cell-free DNA (cfDNA), a highly sensitive, culture-independent method for studying microbial diversity. cfDNA is made up of DNA fragments, typically 50–200 bp long, found in circulation (36). cfDNA is primarily human in origin with a small but rich microbial component (22, 36, 37). With high-throughput sequencing, cfDNA lends itself to noninvasive testing and has transformed diagnostics in perinatal testing and other settings (38–41). Metagenomic sequencing has facilitated the identification of mostly novel phage sequences in the gut (42), blood (22), respiratory tract (43), skin (44), vaginal tract (45), urinary tract (46), and other body sites (47). Numerous studies have demonstrated phage DNA in the cfDNA from pregnant women (22), patients with sepsis (48), organ transplant recipients (49), and patients with cardiovascular disease (50) as well as in infected fluids such as urine, bronchoalveolar lavage fluid, joint fluids, cerebrospinal fluid, and others (51).

To determine whether humans are exposed to bacteriophages or their products in utero, we asked whether bacteriophage DNA could be detected in cfDNA from human umbilical cord blood collected at birth and whether bacteriophage sequences are shared across maternal-infant dyads. In 2 independent cohorts, we identified bacteriophage sequences in cfDNA of paired maternal and umbilical cord blood samples. Our data suggests that the human fetus is exposed to bacteriophage DNA in utero, stimulating questions about how bacteriophages or their products may influence the developing fetus.

## Results

### Bacteriophage DNA is present in human umbilical cord blood.

**Figure 1:**
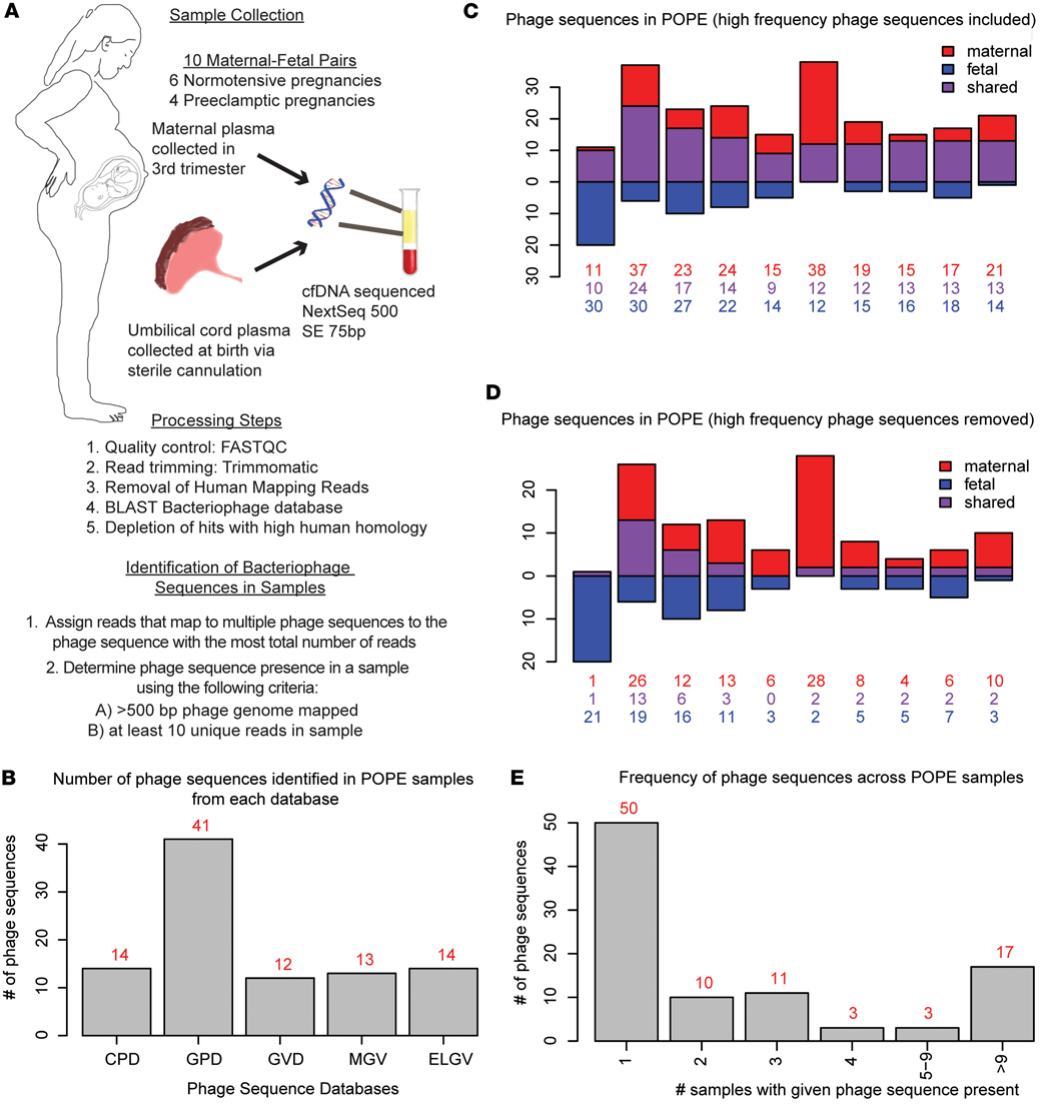
Bacteriophage DNA in umbilical cord blood in POPE dataset. (**A**) Study design, data processing, and analysis. (**B**) Bar graph with the number of phage sequences identified from each phage database among all POPE samples. (**C**) Bar graph of 10 maternal-fetal pairs plotting the number of phage sequences identified only in maternal samples (red), only in fetal samples (blue), or in both samples from each pair (purple). Numbers below represent the number of phage sequences in each category. (**D**) As in **C**, with prevalent phage sequences removed. (**E**) Histogram depicting the number of samples in which each phage sequence was present. Red number indicates the number of phage sequences in each category. **C**–**E** represent the combined results from the 5 phage databases.

To test the hypothesis that the fetus is exposed to bacteriophage signals, we first searched for 474,031 bacteriophage sequences from 5 phage databases in the cfDNA of 10 paired samples of maternal and umbilical cord plasma (Figure 1A), which were part of the Placental Origins of Preeclampsia (POPE) study. Among the 10 pairs, six were from normotensive pregnancies and the remaining four were affected by preeclampsia. The clinical characteristics of these samples are presented in Table 1.

To identify bacteriophage sequences within the metagenomic sequencing data, we applied a phage annotation pipeline using previously published methods, as outlined in Figure 1A. In brief, raw data was quality controlled and trimmed using standard bioinformatics tools, including FASTQC and Trimmomatic. Human reads were subtracted by mapping to the human reference genome GRCh38 via Bowtie2. We then performed an NCBI BLAST search using a Curated Phage Database (CPD) of 26,159 annotated bacteriophage sequences from the NCBI collection (48) and four additional databases of largely unknown and uncharacterized bacteriophage sequences derived from studies of the human gut (Supplemental Table 1; supplemental material available online with this article; https://doi.org/10.1172/jci.insight.183123DS1) (42, 52–54). These include 142,809 bacteriophage sequences identified in human gut metagenomes from across the globe (GPD) (42); 33,242 viral sequences pooled from 1,986 individuals across 33 datasets of human gut studies (GVD) (52); 82,141 viral sequences from infant gut samples (ELGV) (53); and 189,680 viral sequences from 11,810 stool samples (MGV) (54). Reads with significant hits were subjected to a secondary and more stringent human sequence removal, in which all reads with significant BLAST hits to any human sequence deposited in the NCBI nuccore were removed. This was followed by a final BLAST alignment to each of the five databases, allowing reads to map to up to 20 phage genomes per database. Reads that mapped to multiple phage sequences were assigned to the phage that had the highest total number of mapping reads within a database. We counted only those phage sequences with at least 10 unique reads covering at least 500 bp of their genome.

Using these criteria, phage sequences were identified from each of the 5 databases in the POPE samples (Figure 1B and Supplemental Table 2). We identified bacteriophage sequences in all samples from the POPE dataset, with 94 sequences identified across all samples (Figure 1C). In the cord blood samples, 55 phage sequences were identified, and 41 of these were also identified in maternal samples. Seven phage sequences were present in all 20 samples, which could represent phage sequences commonly circulating in blood or contaminants from sample collection or processing (Supplemental Table 3); however, we did not identify any phage sequences meeting our bioinformatic criteria in nuclease free water or PBS samples that were processed via the same methods used here (48). All 7 phage sequences were originally identified in human gut samples and were part of the GPD or GVD databases. To determine the impact of these prevalent phage sequences on the phage signal in our samples, we reanalyzed the data excluding all phage sequences present in more than 75% of our samples. Bacteriophage sequences were identified in all samples, and 16 of 20 samples had 4 or more phage sequences (Figure 1D). No phage sequences were specifically enriched or depleted in preeclamptic samples (Supplemental Table 3).

These data indicate that phage DNA is a common constituent of both maternal and fetal circulations, which is transmitted prenatally in humans from mother to fetus.

### Phage sequences overlap in maternal-infant dyads from the POPE dataset.

We then asked whether phage sequences were present across maternal-infant dyads (samples from the same pregnancy). We observed that 44 of the 94 phage sequences identified in the POPE dataset were present in more than 1 sample and 29 of these were present across at least 1 maternal-infant dyad (Supplemental Table 3).

Across all dyads, we identified phage sequences that were present in a maternal sample but absent in the paired cord blood sample or present in the cord blood but absent in the paired maternal sample (Figure 1, C and D). We found that 9 of 10 dyads had at least 1 shared phage sequence, even after excluding the potential contaminants (Figure 1, C and D). The specific phage sequences that were found in 1 dyad were often found in other dyads (Supplemental Table 4).

We next set out to determine if phage sequences were more likely to match across maternal-infant dyads compared with unrelated samples. Many phage sequences were either rare (present in only 1 sample) or very common and could not be used to discriminate pairings. Thus, we focused on phage sequences that were identified in exactly 2 samples from the POPE dataset and determined the frequency of dyads among these pairs. Ten phage sequences were present in exactly 2 samples from the POPE dataset and 3 of the 10 sample pairs associated with these sequences were dyads compared with 0.5 out of 10 pairs expected to be dyads by random chance, though this was not statistically significant due to the low number of phage sequences identified in exactly 2 samples (Supplemental Figure 1A).

Together, these data indicate that phage DNA in the fetal and maternal circulations may be related to each other, but, given the low coverage and limited sampling of the total phage diversity captured across the five databases, we were not able to detect a strong pattern.

### Unique phage sequences in the POPE dataset.

We next assessed the heterogeneity of the phage sequences in these samples using the presence of unique phage sequences as a read out. To this end, we counted the number of samples containing each phage sequence. Fifty of the 94 identified phage sequences were present in only 1 sample each (Figure 1E). These 50 unique phage sequences were distributed across 6 cord blood samples and 6 maternal samples, with 1–22 unique phage sequences per sample.

These data support the idea that the phageome is individual specific (52, 55–58) and that this individualization of phage populations starts at the earliest points of phage exposure.

### Bacteriophage DNA is present in umbilical cord blood from a second cohort.

**Figure 2:**
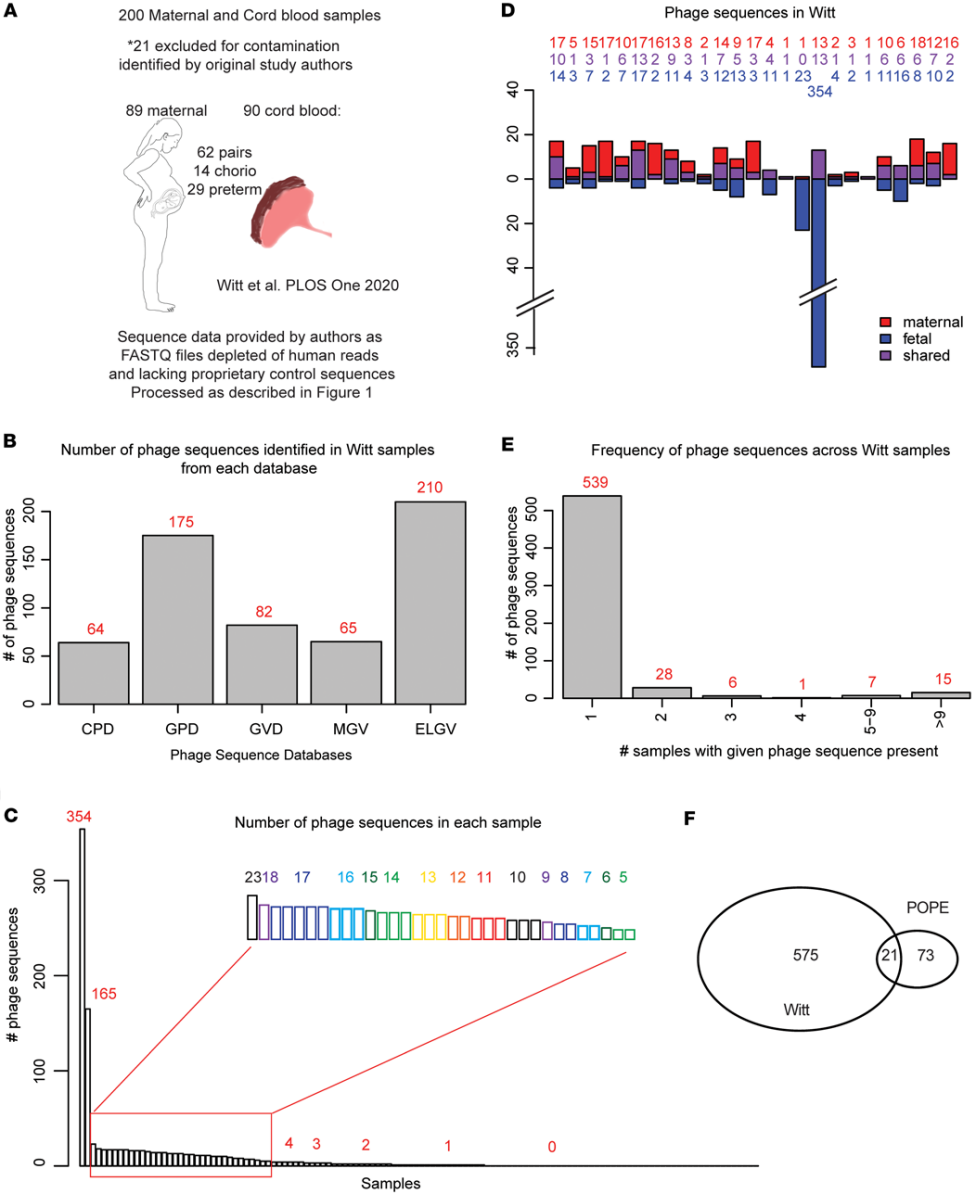
Bacteriophage DNA in umbilical cord blood in Witt dataset. (**A**) Description of the cohort from the Witt dataset. (**B**) Bar graph with the number of phage sequences identified from each phage database among all Witt samples. (**C**) Bar graph of the number of phage sequences (red) present in each sample. (**D**) Bar graph of maternal-fetal pairs in which both samples contained phage sequences. Plot depicts the number of phage sequences identified only in maternal samples (red), only in fetal samples (blue), or in both samples from each pair (purple). Numbers above represent the number of phage sequences in each category. (**E**) Histogram depicting the number of samples in which each phage sequence was present. Red number indicates the number of phage sequences in each category. (**F**) Venn diagram depicting the overlap in the phage sequences identified in the POPE and Witt cohorts. **C**–**F** represent the combined results from the 5 phage databases.

To validate our findings, we searched for bacteriophage DNA in a second dataset of cfDNA from paired maternal and umbilical cord samples originally reported by Witt et al. (59). This dataset contained 89 maternal samples and 111 fetal samples. Twenty-one of the umbilical cord blood samples were identified by the original study authors as likely contaminated based on bacterial sequencing, so these samples were excluded from our analysis (Figure 2A). The remaining samples contained 62 pairs of maternal and umbilical cord blood samples from maternal-infant dyads. Many of these samples came from deliveries affected by chorioamnionitis (14 pairs) or from preterm deliveries (29 pairs). To analyze these data from the Witt dataset, we used the same methods and criteria detailed above for the POPE dataset.

We identified 596 phage sequences from the 5 phage databases across all samples of the Witt dataset (Figure 2B). In the cord blood, 581 phage sequences were identified, and 42 of these were also present in maternal samples (Supplemental Table 5). In this cohort, we found no phage sequences present in greater than 75% of samples. No phage sequences were identified in 50 of 124 samples (Figure 2C). Of the remaining 74 samples, 41 contained 4 or more phage sequences. Phage sequences identified in more than 7 samples were classified by their presence in term, preterm, chorioamnionitis-unaffected and chorioamnionitis-affected samples (Supplemental Table 6). Escherichia virus lambda, elgv_52793, and GVD_30371 sequences appeared to be more prevalent in term than preterm samples. There were 4 phage sequences, MGV-GENOME-0336697, elgv_9621, uvig_539815, and uvig_579209, that were not found in any chorioamnionitis-affected samples but were present in 4 or 5 samples that were chorioamnionitis unaffected.

These data indicate that phage DNA is a common constituent of fetal circulations. The enrichment of phage sequences in term and chorioamnionitis-unaffected samples could indicate a protective effect of these phages or their bacterial hosts against preterm labor or chorioamnionitis. Alternatively, the phages or bacterial hosts associated with the term-enriched sequences may be more prevalent later in pregnancy.

### Phage sequences overlap in maternal-infant dyads in the Witt dataset.

Again, we assessed the overlap of phage sequences across maternal-infant dyads. Fifty-seven phage sequences were identified in more than 1 sample from the Witt dataset, and 33 of these were found across at least 1 maternal-infant dyad (Supplemental Table 5). Among the dyads with bacteriophage DNA present in both maternal and fetal samples, all but 1 dyad had at least 1 shared phage sequence from 1 of the 5 databases (Figure 2D). Eight phage sequences were identified across 4 or more dyads (Supplemental Tables 5 and 7).

To determine whether phage sequences were more likely to be shared between maternal-infant dyads than expected by chance, we again specifically examined the 28 phage sequences that were present across exactly 2 samples from the Witt cohort. We found 11 of the 28 sample pairs associated with these sequences were maternal-infant dyads compared with 0.2 out of 28 pairs expected to be dyads by random chance (*P* value < 0.001, Supplemental Figure 1B).

These data indicate that the overlap in phage sequences across maternal-infant dyads is unlikely to be due to random chance.

### Unique phage sequences in the Witt dataset.

We found that 539 of the 596 identified phage sequences were present in a single sample (Figure 2E). Of these, 494 were identified in 1 of 2 cord blood samples. Nine cord blood samples and 10 maternal samples contained unique phage sequences, with 1–337 unique phage sequences per sample.

### Common phage sequences identified across samples and cohorts.

We identified 21 phage sequences in both cohorts (Table 2 and Figure 2F). The most common phage sequences identified were Lambda phage from *Escherichia*, AcaML1 from *Acidithiobacillus*, phiDP10.3 from *Dickeya*, and the uncharacterized gut phages from the GPD (“uvig_578591”, “uvig_576852”, “uvig_461583”, “uvig_315137”), GVD (“GVD_30371”, “GVD_5336”), and ELGV (“elgv_52793”) databases.

These data indicate that certain phage sequences are prevalent in maternal and fetal circulations.

### Limited characterization of phage sequences identified in cfDNA.

To characterize the phage sequences identified in the POPE and Witt samples, we extracted the known and predicted morphologies and host bacteria genera from the metadata of the published phage databases. Less than 20% of phage sequences identified had associated morphology or host bacteria predictions in their parent databases. The most common known or predicted morphologies were siphoviridae (14 phage sequences) in the POPE dataset, and myoviridae (45 phage sequences), siphoviridae (31 phage sequences), and podoviridae (17 phage sequences) in the Witt dataset.

The most common known or predicted bacterial host genera of identified phage sequences were *Escherichia* (55 phage sequences) and *Streptococcus* (45 phage sequences) (Table 3). Many of the known or predicted bacterial host genera are human gut inhabitants, such as *Escherichia, Enterobacter, Campylobacter, Listeria,* and *Klebsiella*. In addition, we identified 513 phage sequences with unknown bacterial hosts, but which were originally identified in gut samples. In addition to these gut-associated phage sequences, we identified 67 phage sequences with known or predicted bacterial hosts from *Streptococcus, Megasphaera*, *Prevotella*, *Bifidobacterium*, *Roseburia*, *Faecalibacterium*, *Clostridium*, *Ruminococcus*, and *Lactobacillus*, bacterial genera that are also commonly found in the vaginal tract (60–62). Of these 67 phage sequences, 62 appeared exclusively in cord blood samples. Additionally, we identified 2 prevalent phage sequences, *Acidithiobacillus* phage AcaML1 and *Dickeya* phage phi10.3, which were associated with host bacteria that have no known human habitat.

These data indicate that many circulating phage sequences are likely derived from the gut, oral cavity, or vaginal tract, and that the source of the phage sequences in the maternal and fetal circulations may be different.

## Discussion

In this study, we found bacteriophage DNA in umbilical cord blood collected at birth and identical phage sequences in maternal blood, implicating fetal exposure to bacteriophage DNA. We identified numerous phage sequences in maternal and cord blood plasma, many of which were unique to a single sample. This is consistent with previous studies demonstrating that the phageome is individual-specific (52, 55–58). These findings were present across 2 datasets, comprising samples collected at 3 institutions and processed by independent groups. Both datasets included matched maternal and umbilical cord plasma, allowing us to directly compare phage DNA across maternal-infant dyads.

By utilizing cord blood collected at birth, we were able to assess fetal exposure to bacteriophage DNA in humans. In multiple animal models, high titer phages are inefficiently transmitted from mother to fetus during pregnancy (23–29). In humans, the best evidence for prenatal transmission of bacteriophages is the identification of phages and phage DNA in first meconium stools (7, 8, 19). Meconium is formed in utero and is likely sterile (21), but by the time first meconium stools are collected, neonates have been exposed to the outside world and theoretically could have acquired phages or their products postnatally or during delivery. Our study adds to this body of work by demonstrating that the fetus is exposed to bacteriophage DNA in utero.

While we identified bacteriophage DNA in our samples, our methods could not determine whether bioactive phage particles were present. Metagenome sequencing is known to capture a different subset of viruses than virus-like particles (VLP) enrichment, with studies comparing the methodologies in the gut reporting 10%–40% concordance (52, 53). Unlike the gut, plasma has very low phage VLP abundance (35). In fact, Feng et al. isolated VLP from 100 pooled plasma samples to have enough biomass for analysis, an approach that prevents comparison of phage sequences across individual samples (35). A significant limitation of phage identification via short read sequencing is that phage sequences detected may be from fragments rather than intact genomes with biological activity. Thus, these studies require further biological validation. Nevertheless, unencapsidated phage DNA may elicit its own effects on the fetus. Naked phage DNA that was orally ingested by pregnant mice was recovered in the nuclei of cells from various fetal tissues, though the functional significance of this finding is unclear (63). Additionally, phage DNA is sufficient to stimulate immune responses in vitro (64) and may stimulate fetal immune development, as observed with other maternal-derived microbial products (65–67). How phage DNA interacts with maternal and fetal immune systems and the impact of fetal exposure to phage DNA are open questions.

The unique anatomy of the maternal-fetal interface implies a novel route of phage DNA transmission, either transplacental or across fetal membranes. Others have also shown that bacteriophage DNA circulates in maternal blood (22), which directly contacts cytotrophoblasts and syncytiotrophoblasts of the placenta. But phages or their products must cross additional cellular layers in the placenta to access the fetal circulation (68). Alternatively, they could pass to the fetus via the fetal membranes, entering the amniotic fluid, which is then swallowed, providing a route to the fetal gut. While in utero, bacterial colonization is unlikely (69, 70), transient bacterial exposure may be sufficient to transmit phages or phage DNA. Phage DNA could also be passively transmitted from mother to fetus, as likely occurs in mice (63). Intriguingly, phage DNA has also been detected in other immune-restricted sites, such as the CNS (47).

We did not detect phage DNA in nearly half of the samples from the Witt dataset. This likely is due to under sampling or insufficient sequencing depth, but could mean that phage DNA is not present in all fetal circulations; however, we detected phage sequences in all cord blood samples from the POPE dataset. Many of these sequences were unique and thus could not be attributed to systematic contamination from reagents used in sample processing or sequencing. In both cohorts, there were samples with bacteriophage sequences in the maternal blood that were absent in the fetal blood and vice versa. This may be due to timing differences — in the POPE study, maternal blood was collected during a third trimester appointment and neonatal blood was collected at the time of delivery. The phage sequences circulating in maternal blood may be different at those 2 time points. Alternatively, the phage sequences present in the umbilical cord plasma may have transiently circulated in the maternal plasma earlier in pregnancy and then persisted in the fetal blood. Or, maybe there is some selectivity to transmission of phages, their hosts, or their products, perhaps occurring at the level of the placenta, resulting in unequal transmission of phage sequences from mother to fetus.

Another potential explanation for this discrepancy is that maternal blood may not be the source of the phage sequences we identified in cord blood. Our group previously reported that, among asymptomatic healthy adults, the most common bacteriophage hosts represented in plasma were *Escherichia*, *Enterobacter*, *Pseudomonas*, and *Cutibacterium* (48). Park et al. also detected bacterial sequences from *Escherichia*, *Streptococcus, Staphylococcus, Enterococcus,* and *Bacteroides,* as well as other gut anaerobes, in cfDNA samples (71). Most of the phage sequences we identified were associated with gut-derived bacteria, too; however, our data is biased to detect gut phage sequences because we only mapped to known phage sequences from established databases, and 4 of the 5 databases we used were originally derived from gut samples. The source of the phage DNA in the cord blood could alternatively be the vaginal microbiome, though associated phage sequences are underrepresented in our study. Nevertheless, we found that the 67 phage sequences from vagina-associated bacterial hosts were more common in cord blood samples. In this case, the fetus could be exposed to phage products ascending across fetal membranes from the maternal vaginal tract, as observed for some fetal infections (72, 73).

Our ability to accurately identify the phage sequences in our samples is limited by the quality and quantity of the phage sequences deposited in reference databases. The phage sequences in the NCBI database, which were used to assemble the CPD, represent a small subset of the total biodiversity of phages that exist in nature, and a large number of NCBI sequences have been shown to be incorrectly labeled (74). For example, we identified the *Acidithiobacillus* phage AcaML1 in samples across both cohorts; however, the host bacteria *Acidithiobacillus caldus* is an extreme acidophile that has not been described in human specimens (75). Similarly, *Dickeya* is a bacterial genus with no known association with human hosts (76). We think it is unlikely that AcaML1 and *Dickeya* phage phiDP10.3 are the true identities of these phage sequences, and, rather, these phages share sequence homology with uncharacterized phages that infect a human-associated host. Kowarsky et al. also observed sequence homology between their assembled contigs from cfDNA plasma samples and phage sequences from exotic environments (22). In contrast to the CPD, the phage sequences in the GPD, GVD, ELGV, and MGV databases were all derived from human gut samples, but most of the phage sequences are uncharacterized. These 5 databases are largely nonoverlapping; however, we identified 2 pairs of sequences (1st pair: MGV-GENOME-0336697, uvig_539815; 2nd pair: MGV-GENOME-0320117, uvig_473047) that appear to be duplicates between databases based on their presence across the same samples, with identical genome coverage and number of uniquely mapped reads. The characterization of novel phage sequences is a rapidly evolving field, and more work in this area is needed to improve phage annotation.

One potential weakness of cfDNA is the possibility of false positive identifications, given the short size of the DNA fragments involved (77). To avoid this source of error, we took a conservative approach to identifying bacteriophage sequences, only counting those with substantial (greater than 500 bp) genome coverage. Indeed, using these bioinformatic criteria, no phage sequences from the 5 databases were identified in our negative control samples. Yet, this approach likely underestimated the number of bacteriophage sequences in our samples. In addition, due to low genome coverage of cfDNA compared with sequencing studies from gut-derived samples, we were not able to perform more detailed strain level analyses to validate transmission (12, 20). Given the stringency of our methods, the number of phage sequences identified was too small to make any meaningful comparisons of phage number, diversity, morphology, or other phage characteristics between maternal and fetal blood or any other sample categories, though a recent study suggested that the bacteriophages in the maternal gut are differentially abundant in preeclamptic versus normotensive pregnancies (78). Future studies will examine links between phages, bacterial cfDNA, and pregnancy-related outcomes.

## Methods

### Sex as a biological variable.

Sex was not considered a biological variable for this study. Cord blood samples were collected from both males and females, but due to sample availability, our POPE samples were biased to contain more samples from pregnancies with male fetuses. It is not known whether our findings apply equally to males and females.

### Sample collection.

Ten paired maternal and cord blood samples were collected from 6 normotensive and 4 preeclamptic pregnancies. Preeclampsia was defined according to the 2013 ACOG executive summary of Hypertension in Pregnancy (79). Control samples were collected from pregnancies with normal blood pressure and no proteinuria. Enrollment criteria for patients with preeclampsia and normotensive controls included maternal age 15–45, gestational age greater than or equal to 20 weeks, and delivery planned at Lucile Packard Children’s Hospital (Stanford, California, USA). Exclusion criteria included the presence of intraamniotic infection, preterm premature rupture of membranes, known fetal anomaly, and intrauterine fetal demise.

Maternal peripheral blood samples were collected at study enrollment at a third trimester visit via standard venipuncture. At delivery, umbilical cord blood was sampled via sterile cannulation of the umbilical vein following delivery. All samples were collected into EDTA-coated tubes and processed within 1 hour. Samples were centrifuged at 1,200*g* for 10 minutes at 4**°**C to separate plasma from cellular components. Plasma was collected into a fresh tube, and samples were spun again at 2,000*g* for 10 minutes at 4**°**C. Plasma aliquots were then stored at –80**°**C.

### cfDNA isolation/library prep/next-generation sequencing.

Plasma samples of 500 μL aliquots were provided directly to the Stanford Genomics Service Center for DNA isolation, library preparation, and sequencing. Following the standard protocols, DNA was extracted with NucleoSpin cfDNA kit from Takara Bio and libraries were prepared with the KAPA Hyper Kit with overnight ligation at 16 degrees. Samples were subsequently sequenced on the NextSeq 500 platform, as 75 base pair single end reads. Sequencing depth per sample is reported in Supplemental Table 2. 

The dataset from Witt et al. (59) was provided by the authors as raw sequencing reads depleted of human mapping sequences and Karius proprietary control sequences. Of the 200 samples provided, 21 were removed from our analysis based on suspected contamination, as identified by original study authors.

### Read quality control and human read removal.

Raw sequencing reads were subjected to quality control measures and human read removal, as previously described (48). Briefly, the FASTQC software (80) was used to evaluate read quality in preparation for trimming of low-quality reads and adapter sequences with Trimmomatic 0.39 (81). Post-trim read quality was confirmed across samples with MultiQC (82). Bowtie2 v2.4.4 (83) was used to map high quality reads to the human reference genome GRCh38. Unmapped reads were considered potential non-human reads. Using samtools (84), these reads were extracted as a bam file, converted to FASTQ format, and ultimately converted to FASTA format with Seqtkv1.3 (85). A second step to remove sequences with human homology was performed with BLAST v2.13.0 (86, 87). In this step, reads that aligned to the human-associated sequences (taxids 9606, 63221 or 741158) from the NCBI nucleotide database (www.ncbi.nlm.nih.gov/nuccore, as retrieved November 28, 2021) were discarded.

### Bacteriophage sequence mapping.

Cleaned, processed, non-human reads underwent a third BLAST alignment to each of 5 phage databases (Supplemental Table 2) (42, 48, 52–54) with a culling limit of 20, selecting for reads with an e-value below 0.0005. To ensure compatibility with the phage sequence identification pipeline, phage sequences from GVD and ELGV databases were renamed to a sequential numbering system using the following command: ‘seqkit replace -p ‘.+’ -r ‘GVD_{nr}’ GVD.fasta > gvd.fasta’, with GVD replaced by ELGV to rename sequences in the ELGV database. Reads that mapped to multiple phages were assigned to a single phage through an iterative multistep process. First, all phages that were associated with multimapping reads were identified. Within this group, the phage with the highest number of unique mapping reads was identified, and all mapping reads were assigned to this phage. These reads were then removed from the pool and the process was repeated to identify the phage with the next highest number of unique mapping reads, until all reads were assigned to a single phage.

### Identifying phage sequences.

For downstream analysis, phage sequences were identified in each sample as those with at least 10 unique reads that mapped across at least 500 bp of the phage genome. Unique reads were defined as those with unique sequencing IDs and unique start sites in the phage genome. To calculate genome coverage, a sequential numeric vector was created for each phage genome from 1 to the length of the genome. Then, the genome indices of each mapping read were subtracted from the vector, leaving a vector that contained only the positions of the nonmapped phage genome. The length of the nonmapped phage genome vector was subtracted from the length of the total phage genome to calculate base pair coverage. Bacterial host identity and phage morphology were extracted from the metadata of the published phage databases as available.

### Statistics.

Statistics were performed in RStudio 2023.06.1+524. For BLAST analysis an E-value cutoff of 0.0005 was used to account for multiple comparisons. Fisher’s exact test was used to compare observed-to-expected frequency of dyads among pairs of samples containing a shared phage sequence. A *P* value less than 0.05 was considered significant.

### Study approval.

Written informed consent was obtained prior to participation from all participants involved in the study. The study was conducted in accordance with the Declaration of Helsinki and approved by the Institutional Review Board of Stanford University (protocol 34745, original date of approval August 31, 2020, and continuously renewed through the time of this manuscript submission).

### Data availability statement.

Sequencing data with human reads removed have been deposited into NCBI SRA under bioproject PRJNA1095563. R scripts for data processing and plot generation are available at: https://doi.org/10.5281/zenodo.10946723 Supporting data values for all figures are provided in an excel file.

## Author contributions

JAS, PLB, and VDW conceptualized the study. Sample collection and processing was performed by PN and NM and was supervised by VDW. Methodology was developed by JAS, NLH, and LJB. JAS performed the investigation and data analysis. PMG prepared tables for the manuscript. JAS drafted the manuscript, and JAS, NM, PLB, and VDW edited the manuscript. All authors have read and agreed to the published version of the manuscript.

## Supplementary Material

Supplemental data

Supporting data values

## Figures and Tables

**Table 1:**
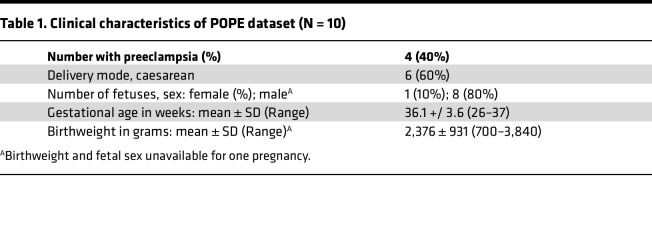
Clinical characteristics of POPE dataset (N = 10)

**Table 2:**
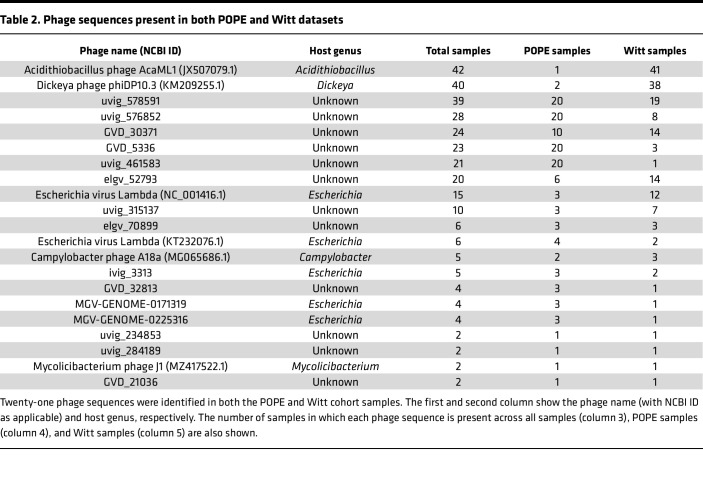
Phage sequences present in both POPE and Witt datasets

**Table 3:**
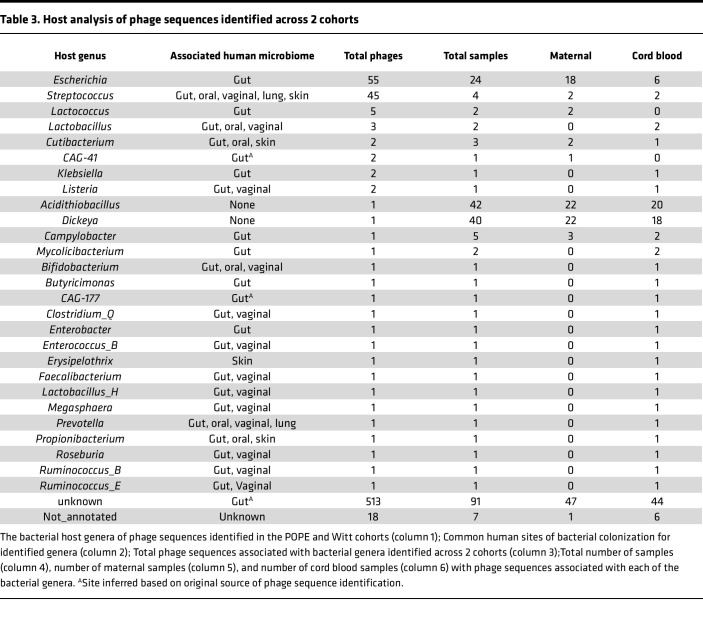
Host analysis of phage sequences identified across 2 cohorts

## Version 1 07/08/2025

Electronic publication
