# Supplementary material for: Identification of bacteriophage DNA in human umbilical cord blood
Source: JCI Insight. 2025 Jul 8;10(13):e183123. doi: 10.1172/jci.insight.183123 (PMC12288903; doi:10.1172/jci.insight.183123)
Supplement: Supplemental data [file jciinsight-10-183123-s201.pdf]

Supplemental Table 1: Phage Databases

Database	Source	# of Sequences	Reference
Curated Phage Database (CPD)	NCBI sequences	26,159	Haddock et al.(48)
Gut Phage Database (GPD)	Human Gut (28,060 metagenomes, globally distributed)	142,809	Camarillo et al.(42)
Gut Virome Database (GVD)	Human Gut Metagenome (33 datasets, 2697 samples, 1986 individuals)	33,242	Gregory et al.(52)
Early-Life Gut Virome (ELGV)	Human Infant Gut (8130 fecal metagenomes < 3yr)	82,141	Zeng et al.(53)
Metagenomic Gut Virus catalogue (MGV)	Human Gut (11,810 stool samples)	189,680	Nayfach et al.(54)

Supplemental Table 2: POPE Sequencing

Sample	Total Reads	Non-human	CPD	GPD	GVD	ELGV	MGV
**1_POPE_MB**	34,133,139	1,298,053	2,360 (2)	22,400 (22)	877 (7)	68,513 (3)	18,783 (3)
**1_POPE_CB**	32,978,123	1,308,084	718 (2)	6,838 (18)	731 (6)	20,491 (3)	3,408 (1)
**2_POPE_MB**	36,867,427	1,145,333	550 (0)	10,796 (20)	310 (3)	33,791 (0)	5,241 (0)
**2_POPE_CB**	30,021,651	1,021,650	2,888 (3)	12,203 (17)	1,395 (5)	70,605 (1)	4,312 (1)
**3_POPE_MB**	35,879,971	836,315	2,328 (2)	27,685 (17)	295 (2)	80,727 (2)	23,344 (1)
**3_POPE_CB**	18,583,685	657,620	5,811 (1)	10,164 (17)	3,305 (3)	18,825 (0)	6,989 (1)
**4_POPE_MB**	20,569,035	423,779	498 (0)	6,297 (14)	128 (1)	20,901 (0)	3,504 (0)
**4_POPE_CB**	21,202,765	571,187	982 (0)	3,840 (14)	219 (2)	11,244 (0)	1,124 (0)
**5_POPE_MB**	21,295,621	453,396	933 (1)	10,712 (16)	407 (2)	20,038 (1)	7,442 (1)
**5_POPE_CB**	18,029,634	324,915	212 (0)	4,875 (13)	85 (1)	6,555 (0)	2,144 (0)
**6_POPE_MB**	18,384,841	420,218	628 (0)	3,743 (14)	196 (1)	10,787 (1)	1,573 (1)
**6_POPE_CB**	14,597,182	314,745	1,014 (1)	22,096 (13)	210 (2)	82,920 (1)	16,957 (1)
**7_POPE_MB**	20,907,878	423,045	3,049 (4)	5,617 (17)	326 (5)	17,999 (6)	4,077 (6)
**7_POPE_CB**	19,648,144	473,924	862 (0)	4,756 (11)	173 (1)	17,549 (0)	2,231 (0)
**8_POPE_MB**	21,629,774	510,159	991 (1)	18,134 (15)	167 (2)	54,225 (1)	13,686 (0)
**8_POPE_CB**	17,089,816	423,196	268 (0)	2,791 (14)	120 (1)	4,384 (0)	813 (0)
**9_POPE_MB**	17,062,306	305,501	65 (0)	1,619 (10)	65 (1)	335 (0)	169 (0)
**9_POPE_CB**	20,105,070	476,999	1,441 (3)	9,494 (16)	657 (4)	52,262 (5)	5,278 (2)
**10_POPE_MB**	14,568,442	403,189	1,545 (1)	91,662 (11)	178 (2)	170,622 (0)	84,699 (1)
**10_POPE_CB**	15,135,183	484,594	628 (0)	17,871 (13)	119 (1)	82,340 (0)	15,705 (0)
**PBS**	5220	3924	706 (0)	366 (0)	21 (0)	660 (0)	24 (0)
**H2O**	5690	3472	1,341 (0)	1,095 (0)	31 (0)	1357 (0)	25 (0)

For the twenty POPE samples and two negative control samples, across the columns are listed total reads (column 2), non-human reads (column 3), followed by the number of reads mapped to each phage database with the number of phage sequences that met criteria of 10 unique reads and 500 bp genome coverage, listed in parentheses.

Supplemental Table 3: Characteristics of all phage sequences identified in POPE samples

Phage Name (NCBI ID)	Total POPE	Maternal	Cord Blood	Dyad	NT	PE	Host	Morphology	Mean # Reads Per Sample (min, max)	Mean BP Genome Coverage (min, max)
uvig_461583	20	10	10	10	12	8	unknown	unknown	278 (161, 470)	4682 (3684, 5935)
uvig_576852	20	10	10	10	12	8	unknown	unknown	58 (41, 89)	2843 (2162, 3640)
uvig_578591	20	10	10	10	12	8	unknown	unknown	39 (21, 72)	1988 (1219, 2997)
GVD_5336	20	10	10	10	12	8	unknown	unknown	34 (23, 55)	1551 (1213, 2068)
uvig_576872	20	10	10	10	12	8	unknown	unknown	27 (14, 48)	1693 (938, 2985)
uvig_576406	20	10	10	10	12	8	unknown	unknown	27 (12, 45)	1680 (833, 2424)
uvig_576397	20	10	10	10	12	8	unknown	unknown	23 (12, 42)	1467 (757, 2643)
uvig_576871	19	9	10	9	12	7	unknown	unknown	29 (17, 43)	1825 (1025, 2499)
uvig_576859	19	9	10	9	12	7	unknown	unknown	26 (12, 45)	1574 (900, 2273)
uvig_461631	17	9	8	8	11	6	unknown	unknown	102 (13, 151)	3029 (822, 3894)
uvig_576860	17	9	8	8	10	7	unknown	unknown	18 (12, 29)	1100 (748, 1735)
uvig_461436	15	7	8	5	9	6	unknown	unknown	42 (17, 69)	2086 (1156, 3091)
uvig_461438	15	7	8	6	9	6	unknown	unknown	20 (11, 42)	674 (515, 869)
uvig_578594	14	9	5	4	10	4	unknown	unknown	14 (10, 20)	868 (616, 1132)
GVD_30371	10	5	5	1	6	4	unknown	unknown	20 (10, 41)	1343 (641, 2692)
uvig_461634	10	6	4	3	6	4	unknown	unknown	19 (11, 42)	993 (507, 2011)

uvig_461615	10	4	6	2	5	5	unknown	unknown	16 (10, 23)	927 (589, 1223)
elgv_52793	6	3	3	1	4	2	unknown	unknown	17 (10, 27)	1260 (753, 1955)
uvig_576864	6	4	2	1	2	4	unknown	unknown	12 (11, 16)	723 (596, 976)
uvig_461396	5	2	3	1	2	3	unknown	unknown	11 (10, 13)	674 (579, 735)
GVD_451	4	2	2	1	2	2	unknown	unknown	75 (22, 183)	2421 (1231, 3884)
Escherichia virus Lambda (KT232076.1)	4	3	1	0	2	2	*Escherichia*	Siphoviridae	17 (10, 36)	1139 (594, 2398)
GVD_20433	4	2	2	1	2	2	unknown	unknown	14 (11, 16)	689 (521, 794)
Escherichia virus Lambda (NC_001416.1)	3	1	2	0	2	1	*Escherichia*	Siphoviridae	24 (16, 32)	1735 (1146, 2363)
MGV-GENOME-0225316	3	1	2	0	1	2	*Escherichia*	unknown	18 (14, 21)	969 (749, 1264)
elgv_70899	3	1	2	1	2	1	unknown	unknown	18 (13, 23)	868 (740, 983)
uvig_315137	3	1	2	1	2	1	unknown	unknown	14 (12, 17)	961 (825, 1116)
ivig_3313	3	1	2	0	1	2	*Escherichia*	unknown	13 (12, 16)	684 (600, 767)
GVD_19168	3	0	3	0	1	2	unknown	unknown	13 (10, 18)	767 (657, 966)
uvig_461392	3	1	2	0	2	1	unknown	unknown	12 (10, 14)	796 (705, 956)
elgv_43211	3	1	2	1	2	1	unknown	unknown	12 (11, 14)	910 (817, 1004)
MGV-GENOME-0369678	3	1	2	1	2	1	unknown	unknown	12 (10, 13)	785 (737, 830)
MGV-GENOME-0171319	3	2	1	0	1	2	*Escherichia*	unknown	11 (11, 12)	711 (594, 795)
GVD_32813	3	1	2	0	2	1	unknown	unknown	11 (10, 11)	772 (740, 834)
uvig_576399	2	1	1	0	1	1	unknown	unknown	19 (19, 19)	1075 (1012, 1138)

uvig_578600	2	1	1	0	2	0	unknown	unknown	16<br>(11, 20)	629<br>(501, 757)
elgv_42737	2	1	1	1	2	0	unknown	unknown	15<br>(13, 17)	1106<br>(944, 1267)
Campylobacter phage A18a (MG065686.1)	2	1	1	0	0	2	*Campylobacter*	Not Characterized	14<br>(11, 17)	1021<br>(751, 1291)
uvig_394185	2	2	0	0	2	0	*Cutibacterium*	Siphoviridae	13<br>(12, 14)	962<br>(894, 1029)
Dickeya phage phiDP10.3 (KM209255.1)	2	1	1	0	1	1	*Dickeya*	Ackermannviridae	13<br>(12, 13)	728<br>(586, 869)
GVD_386	2	1	1	1	0	2	unknown	unknown	12<br>(11, 13)	563<br>(515, 610)
GVD_20415	2	1	1	1	2	0	unknown	unknown	12<br>(11, 12)	568<br>(533, 602)
uvig_576854	2	1	1	0	1	1	unknown	unknown	11<br>(10, 11)	621<br>(618, 623)
uvig_576856	2	2	0	0	1	1	unknown	unknown	10<br>(10, 10)	681<br>(657, 704)
Lactococcus phage 49801 (KX160205.1)	1	1	0	0	1	0	*Lactococcus*	Siphoviridae	68 (NA)	4515 (NA)
GVD_22169	1	1	0	0	1	0	unknown	unknown	45 (NA)	3082 (NA)
elgv_26511	1	1	0	0	1	0	unknown	unknown	45 (NA)	3088 (NA)
Streptococcus phage SW1151(MH892376.1)	1	1	0	0	0	1	*Streptococcus*	Siphoviridae	44 (NA)	3291 (NA)
uvig_568874	1	1	0	0	1	0	unknown	Siphoviridae	42 (NA)	2899 (NA)
uvig_357003	1	1	0	0	0	1	unknown	Siphoviridae	37 (NA)	2777 (NA)
MGV-GENOME-0211632	1	1	0	0	1	0	*Streptococcus*	unknown	34 (NA)	2341 (NA)
elgv_71815	1	1	0	0	0	1	unknown	unknown	29 (NA)	2174 (NA)
uvig_188395	1	1	0	0	1	0	unknown	unknown	28 (NA)	1798 (NA)
MGV-GENOME-0243380	1	1	0	0	0	1	*Streptococcus*	unknown	25 (NA)	1848 (NA)
MGV-GENOME-4414956	1	1	0	0	1	0	*Lactococcus*	unknown	21 (NA)	1288 (NA)
uvig_419422	1	1	0	0	1	0	unknown	unknown	20 (NA)	1337 (NA)
uvig_579098	1	1	0	0	0	1	unknown	unknown	19 (NA)	688 (NA)
elgv_59172	1	1	0	0	1	0	unknown	unknown	19 (NA)	1291 (NA)
Lactococcus phage TP901-1 (NC_002747.1)	1	1	0	0	1	0	*Lactococcus*	Siphoviridae	18 (NA)	1256 (NA)
uvig_394472	1	0	1	0	1	0	*Cutibacterium*	Siphoviridae	17 (NA)	1267 (NA)

Streptococcus phage SW19 (MH892367.1)	1	1	0	0	1	0	*Streptococcus*	Siphoviridae	17 (NA)	1238 (NA)
MGV-GENOME-0207849	1	0	1	0	1	0	unknown	unknown	17 (NA)	1267 (NA)
MGV-GENOME-0163115	1	1	0	0	1	0	unknown	unknown	17 (NA)	1125 (NA)
uvig_113176	1	1	0	0	1	0	unknown	unknown	16 (NA)	1042 (NA)
GVD_28885	1	1	0	0	1	0	unknown	unknown	16 (NA)	1041 (NA)
Acidithiobacillus phage AcaML1 (JX507079.1)	1	0	1	0	0	1	*Acidithiobacillus*	Myoviridae	16 (NA)	1133 (NA)
Stx1a-converting phage Stx1_1380 (LC645433.1)	1	0	1	0	0	1	Not_annotated	Siphoviridae	15 (NA)	677 (NA)
MGV-GENOME-0206370	1	1	0	0	1	0	unknown	unknown	14 (NA)	1034 (NA)
Lactococcus phage BIM BV-114 (MZ955869.1)	1	1	0	0	1	0	*Lactococcus*	Siphoviridae	14 (NA)	930 (NA)
elgv_75914	1	1	0	0	1	0	unknown	unknown	14 (NA)	965 (NA)
elgv_3660	1	1	0	0	1	0	unknown	unknown	14 (NA)	1005 (NA)
uvig_461437	1	1	0	0	0	1	unknown	unknown	13 (NA)	704 (NA)
uvig_440553	1	0	1	0	0	1	unknown	unknown	13 (NA)	703 (NA)
uvig_284189	1	1	0	0	1	0	unknown	unknown	13 (NA)	706 (NA)
Propionibacterium phage pa35 (MG820643.1)	1	0	1	0	1	0	*Propionibacterium*	Siphoviridae	13 (NA)	974 (NA)
Mycolicibacterium phage J1 (MZ417522.1)	1	0	1	0	1	0	*Mycolicibacterium*	Podoviridae	13 (NA)	712 (NA)
MGV-GENOME-0224831	1	1	0	0	1	0	unknown	unknown	13 (NA)	745 (NA)
ivig_2065	1	0	1	0	0	1	*Ruminococcus*_B	unknown	13 (NA)	591 (NA)
GVD_21036	1	1	0	0	1	0	unknown	unknown	13 (NA)	644 (NA)
Stx1a-converting phage Stx1_120412 (LC645434.1)	1	0	1	0	0	1	unknown	Siphoviridae	12 (NA)	602 (NA)
elgv_33668	1	0	1	0	0	1	unknown	unknown	12 (NA)	833 (NA)
uvig_473047	1	1	0	0	1	0	*CAG-41*	unknown	11 (NA)	537 (NA)
MGV-GENOME-0320117	1	1	0	0	1	0	*CAG-41*	unknown	11 (NA)	537 (NA)
MGV-GENOME-0201975	1	1	0	0	1	0	unknown	unknown	11 (NA)	816 (NA)
GVD_30336	1	1	0	0	1	0	unknown	unknown	11 (NA)	809 (NA)
elgv_70574	1	1	0	0	0	1	unknown	unknown	11 (NA)	791 (NA)
uvig_90677	1	1	0	0	0	1	unknown	unknown	10 (NA)	545 (NA)
uvig_576870	1	0	1	0	1	0	unknown	unknown	10 (NA)	638 (NA)
uvig_461353	1	1	0	0	0	1	unknown	unknown	10 (NA)	689 (NA)
uvig_234853	1	0	1	0	1	0	unknown	unknown	10 (NA)	681 (NA)

MGV-GENOME-0125413	1	1	0	0	1	0	*Streptococcus*	unknown	10 (NA)	643 (NA)
elgv_53527	1	0	1	0	0	1	unknown	unknown	10 (NA)	600 (NA)
elgv_48824	1	1	0	0	1	0	unknown	unknown	10 (NA)	679 (NA)
elgv_45199	1	1	0	0	1	0	unknown	unknown	10 (NA)	727 (NA)

List of all 94 phage sequences identified in the POPE samples. The first column shows the phage name and NCBI ID (if applicable). The number of samples in which each phage sequence is present across all POPE samples (column 2), maternal samples (column 3), cord blood samples (column 4), and across maternal-infant dyads (column 5), normotensive samples (column 6), and preeclamptic samples (column 7). Known or predicted phage host and morphology are listed when available from prior studies. The last two columns represent the number of unique reads attributed to each phage sequence averaged across all samples in which the phage sequence was identified, and the number of base pairs of the phage genome covered averaged across all of these samples, rounded to the nearest whole number. For both metrics, the range is presented in parentheses, when applicable.

Supplemental Table 4: Phage sequences present across each pair (POPE)

Pair Name	Shared Phage #	List of Shared Phage
1_POPE	24	uvig_315137, uvig_461436, uvig_461438, uvig_461583, uvig_461631, uvig_461634, uvig_576397, uvig_576406, uvig_576859, uvig_576860, uvig_576871, uvig_576872, uvig_578591, uvig_578594, uvig_576852, MGV-GENOME-0369678, elgv_43211, elgv_70899, elgv_52793, GVD_20415, GVD_20433, GVD_30371, GVD_451, GVD_5336
2_POPE	17	uvig_461396, uvig_461436, uvig_461438, uvig_461583, uvig_461615, uvig_461631, uvig_461634, uvig_576397, uvig_576406, uvig_576859, uvig_576860, uvig_576871, uvig_576872, uvig_578591, uvig_576852, GVD_386, GVD_5336
3_POPE	14	uvig_461436, uvig_461438, uvig_461583, uvig_461631, uvig_576397, uvig_576406, uvig_576859, uvig_576860, uvig_576864, uvig_576871, uvig_576872, uvig_578591, uvig_576852, GVD_5336
4_POPE	13	uvig_461438, uvig_461583, uvig_461631, uvig_576397, uvig_576406, uvig_576859, uvig_576860, uvig_576871, uvig_576872, uvig_578591, uvig_578594, uvig_576852, GVD_5336
5_POPE	13	uvig_461438, uvig_461583, uvig_461631, uvig_461634, uvig_576397, uvig_576406, uvig_576859, uvig_576860, uvig_576871, uvig_576872, uvig_578591, uvig_576852, GVD_5336
6_POPE	13	uvig_461436, uvig_461583, uvig_461631, uvig_576397, uvig_576406, uvig_576859, uvig_576860, uvig_576871, uvig_576872, uvig_578591, uvig_576852, elgv_42737, GVD_5336
7_POPE	12	uvig_461436, uvig_461583, uvig_461631, uvig_576397, uvig_576406, uvig_576859, uvig_576871, uvig_576872, uvig_578591, uvig_578594, uvig_576852, GVD_5336
8_POPE	12	uvig_461438, uvig_461583, uvig_576397, uvig_576406, uvig_576859, uvig_576860, uvig_576871, uvig_576872, uvig_578591, uvig_578594, uvig_576852, GVD_5336
9_POPE	10	uvig_461583, uvig_461615, uvig_576397, uvig_576406, uvig_576859, uvig_576871, uvig_576872, uvig_578591, uvig_576852, GVD_5336
10_POPE	9	uvig_461583, uvig_461631, uvig_576397, uvig_576406, uvig_576852, uvig_576860, uvig_576872, uvig_578591, GVD_5336

The number and identities of the shared phage sequences are listed for each of the ten maternal-infant dyads from the POPE cohort.

Supplemental Table 5: Characteristics of all phage sequences present in (>1) Witt sample

Phage Name (NCBI ID)	Total Witt	Maternal	Cord Blood	Dyad	Host	Morphology	Mean # Reads Per Sample (min, max)	Mean BP Genome Coverage (min, max)
Acidithiobacillus phage AcaML1 (JX507079.1)	41	22	19	19	*Acidithiobacillus*	Myoviridae	23 (10, 42)	1399 (567, 2510)
Dickeya phage phiDP10.3 (KM209255.1)	38	21	17	11	*Dickeya*	Ackermannviridae	23 (10, 316)	840 (505, 3735)
elgv_9775	33	19	14	14	unknown	unknown	23 (10, 41)	1322 (563, 2276)
GVD_32950	31	17	14	12	unknown	unknown	19 (10, 33)	963 (546, 1474)
uvig_456227	30	17	13	8	unknown	unknown	15 (10, 44)	702 (519, 1095)
elgv_4493	29	19	10	9	unknown	unknown	18 (11, 31)	1037 (681, 1530)
elgv_76814	26	13	13	7	unknown	unknown	15 (10, 21)	681 (514, 938)
uvig_578591	19	5	14	1	unknown	unknown	15 (10, 26)	793 (618, 1097)
elgv_5160	18	10	8	4	unknown	unknown	12 (10, 15)	713 (570, 909)
elgv_5153	14	8	6	2	unknown	unknown	14 (10, 20)	787 (535, 1124)
elgv_52793	14	13	1	1	unknown	unknown	16 (10, 24)	1051 (667, 1555)
GVD_30371	14	13	1	1	unknown	unknown	22 (12, 39)	1411 (792, 2425)
MGV-GENOME-0336697	12	9	3	1	unknown	unknown	16 (11, 28)	1020 (603, 1802)
Escherichia virus Lambda (NC_001416.1)	12	11	1	1	*Escherichia*	Siphoviridae	23 (13, 39)	1472 (879, 2428)
uvig_539815	12	9	3	1	unknown	unknown	16 (11, 28)	1020 (603, 1802)
uvig_174747	8	6	2	2	unknown	unknown	13 (10, 16)	589 (535, 676)

uvig_576852	8	0	8	0	unknown	unknown	17 (13, 23)	788 (638, 994)
elgv_9621	7	3	4	2	unknown	unknown	17 (12, 27)	1137 (767, 1852)
uvig_315137	7	3	4	1	unknown	unknown	12 (10, 15)	760 (669, 932)
uvig_579209	7	4	3	1	unknown	unknown	11 (10, 17)	671 (545, 825)
elgv_10947	6	3	3	0	unknown	unknown	13 (11, 15)	720 (590, 856)
elgv_6426	6	4	2	1	unknown	unknown	12 (10, 17)	667 (506, 830)
elgv_14246	4	3	1	0	unknown	unknown	12 (10, 13)	621 (613, 624)
elgv_6427	3	1	2	0	unknown	unknown	11 (10, 12)	688 (636, 790)
elgv_70899	3	2	1	0	unknown	unknown	14 (13, 15)	638 (583, 712)
GVD_31891	3	2	1	1	unknown	unknown	13 (12, 15)	847 (749, 1003)
GVD_32763	3	2	1	0	unknown	unknown	11 (10, 12)	647 (575, 738)
GVD_5336	3	0	3	0	unknown	unknown	13 (10, 15)	535 (519, 557)
Campylobacter phage A18a (MG065686.1)	3	2	1	1	*Campylobacter*	unknown	583 (10, 1695)	27368 (634, 78701)
elgv_21268	2	0	2	0	unknown	unknown	65 (11, 119)	1157 (573, 1740)
elgv_22933	2	1	1	0	unknown	unknown	11 (10, 11)	573 (536, 609)
elgv_35865	2	0	2	0	unknown	unknown	95 (12, 178)	1277 (722, 1832)
elgv_52080	2	0	2	0	unknown	unknown	59 (17, 101)	1280 (1130, 1429)
elgv_52855	2	1	1	0	unknown	unknown	23 (20, 25)	1465 (1276, 1654)
elgv_53823	2	0	2	0	unknown	unknown	240 (13, 466)	1420 (769, 2070)

elgv_6429	2	1	1	0	unknown	unknown	14 (10, 17)	764 (550, 977)
GVD_22286	2	1	1	1	unknown	unknown	104 (11, 197)	5796 (664, 10927)
GVD_32530	2	2	0	0	unknown	unknown	11 (10, 11)	689 (662, 716)
GVD_32594	2	2	0	0	unknown	unknown	11 (10, 12)	778 (658, 898)
GVD_6808	2	1	1	1	unknown	unknown	281 (22, 539)	13891 (1337, 26445)
ivig_3313	2	1	1	1	*Escherichia*	unknown	16 (11, 20)	960 (697, 1222)
Escherichia virus Lambda (KT232076.1)	2	2	0	0	*Escherichia*	Siphoviridae	13 (12, 13)	866 (792, 939)
MGV-GENOME-0242084	2	0	2	0	*Streptococcus*	unknown	39 (10, 67)	557 (533, 580)
MGV-GENOME-0245874	2	0	2	0	*Lactobacillus*	unknown	40 (10, 69)	2502 (677, 4327)
Streptococcus satellite phage Javan333 (MK448436.1)	2	0	2	0	*Streptococcus*	Siphoviridae	607 (10, 1203)	6155 (618, 11691)
Bacteriophage sp. (MT835687.1)	2	1	1	0	unknown	unknown	11 (11, 11)	804 (793, 815)
Escherichia phage Lambda_ev243 (NC_049955.1)	2	1	1	1	*Escherichia*	Siphoviridae	19 (13, 25)	1160 (770, 1549)
uvig_116426	2	1	1	1	unknown	unknown	171 (13, 328)	8740 (845, 16635)
uvig_247348	2	1	1	1	unknown	unknown	149 (13, 284)	7950 (862, 15037)
uvig_287054	2	1	1	1	unknown	unknown	121 (14, 227)	6907 (806, 13007)
uvig_316437	2	1	1	1	unknown	unknown	229 (15, 443)	11729 (894, 22563)
uvig_323562	2	1	1	1	unknown	unknown	137 (12, 262)	6920 (737, 13103)
uvig_370894	2	0	2	0	unknown	unknown	649 (74, 1223)	9041 (4849, 13232)
uvig_385029	2	1	1	1	*Escherichia*	unknown	184 (10, 357)	9776 (621, 18931)

uvig_396317	2	0	2	0	unknown	unknown	91<br>(10, 171)	1569<br>(662, 2476)
uvig_577220	2	1	1	1	unknown	unknown	185<br>(10, 360)	9171<br>(664, 17677)
uvig_578399	2	0	2	0	unknown	Myoviridae	276<br>(10, 542)	3557<br>(581, 6532)

List of 57 phage sequences identified in more than one sample from the Witt cohort. The first column shows the phage name and NCBI ID (if applicable). The number of samples in which each phage sequence is present across all Witt samples (column 2), maternal samples (column 3), cord blood samples (column 4), and across maternal-infant dyads (column 5). Known or predicted phage host and morphology are listed when available from prior studies. The last two columns represent the number of unique reads attributed to each phage averaged across all samples, and the number of base pairs of the phage genome covered averaged across all samples, rounded to the nearest whole number. For both metrics, the range across all samples is presented in parentheses.

Supplemental Table 6: Characterization of prevalent Witt phage sequences by clinical features

Phage Name (NCBI ID)	Total Witt	Term	Preterm	No Chorio	Chorio
Acidithiobacillus phage AcaML1 (JX507079.1)	41	24	17	17	4
Dickeya phage phiDP10.3 (KM209255.1)	38	24	14	11	9
elgv_9775	33	19	14	14	2
GVD_32950	31	19	12	13	1
uvig_456227	30	17	13	12	3
elgv_4493	29	17	12	13	1
elgv_76814	26	19	7	8	1
uvig_578591	19	8	11	7	5
elgv_5160	18	12	6	7	1
elgv_5153	14	9	5	5	1
elgv_52793	14	13	1	1	2
GVD_30371	14	13	1	1	2
MGV-GENOME-0336697	12	9	3	4	0
Escherichia virus Lambda (NC_001416.1)	12	11	1	1	1
uvig_539815	12	9	3	4	0
uvig_174747	8	6	2	3	1
uvig_576852	8	3	5	2	3
elgv_9621	7	3	4	5	0
uvig_579209	7	3	4	5	0
uvig_315137	7	3	4	3	1

List of twenty phage sequences identified in seven or more samples from the Witt cohort. The first column shows the phage name and NCBI ID (if applicable). The number of samples in which each phage is present across all Witt samples (column 2), term samples (column 3), preterm samples (column 4), chorio-unaffected samples (column 5), and chorio-affected samples (column 6).

Supplemental Table 7: Phage sequences present across each pair (Witt)

Pair Name	Shared Phage #	List of Shared Phage
1_Witt	13	phiDP10.3, Lambda_ev243, Campylobacter phage A18a, uvig_116426, uvig_247348, uvig_287054, uvig_323562, uvig_385029, uvig_577220, uvig_316437, ivig_3313, GVD_6808, GVD_22286
2_Witt	13	AcaML1, phiDP10.3, uvig_174747, uvig_456227, uvig_539815, MGV-GENOME-0336697, elgv_4493, elgv_5153, elgv_5160, elgv_76814, elgv_9775, GVD_31891, GVD_32950
3_Witt	10	AcaML1, phiDP10.3, uvig_174747, uvig_456227, elgv_4493, elgv_5160, elgv_6426, elgv_76814, elgv_9775, GVD_32950
4_Witt	9	AcaML1, phiDP10.3, uvig_456227, elgv_4493, elgv_5153, elgv_5160, elgv_76814, elgv_9775, GVD_32950
5_Witt	7	AcaML1, phiDP10.3, uvig_456227, elgv_4493, elgv_76814, elgv_9775, GVD_32950
6_Witt	7	AcaML1, phiDP10.3, uvig_456227, elgv_4493, elgv_76814, elgv_9775, GVD_32950
7_Witt	6	AcaML1, phiDP10.3, uvig_579209, elgv_5160, elgv_9775, GVD_32950
8_Witt	6	AcaML1, phiDP10.3, uvig_456227, elgv_4493, elgv_9775, GVD_32950
9_Witt	6	AcaML1, Lambda, elgv_9775, elgv_52793, GVD_30371, GVD_32950
10_Witt	6	AcaML1, phiDP10.3, elgv_4493, elgv_76814, elgv_9775, GVD_32950
11_Witt	5	AcaML1, phiDP10.3, uvig_456227, elgv_4493, elgv_9775
12_Witt	4	AcaML1, elgv_4493, elgv_9775, GVD_32950
13_Witt	3	AcaML1, phiDP10.3, elgv_9775
14_Witt	3	AcaML1, uvig_456227, elgv_9775
15_Witt	3	AcaML1, elgv_76814, GVD_32950
16_Witt	2	AcaML1, GVD_32950
17_Witt	2	AcaML1, elgv_9775
18_Witt	1	AcaML1
19_Witt	1	AcaML1
20_Witt	1	AcaML1
21_Witt	1	uvig_578591
22_Witt	1	uvig_315317
23_Witt	1	elgv_9621
24_Witt	1	elgv_9621

There were 24 maternal-infant dyads from the Witt cohort with shared phage sequences. The number and identities of the shared phage sequences are listed for each pair.

Supplemental Figure 1: Dyad frequency of phage sequences present in two samples.

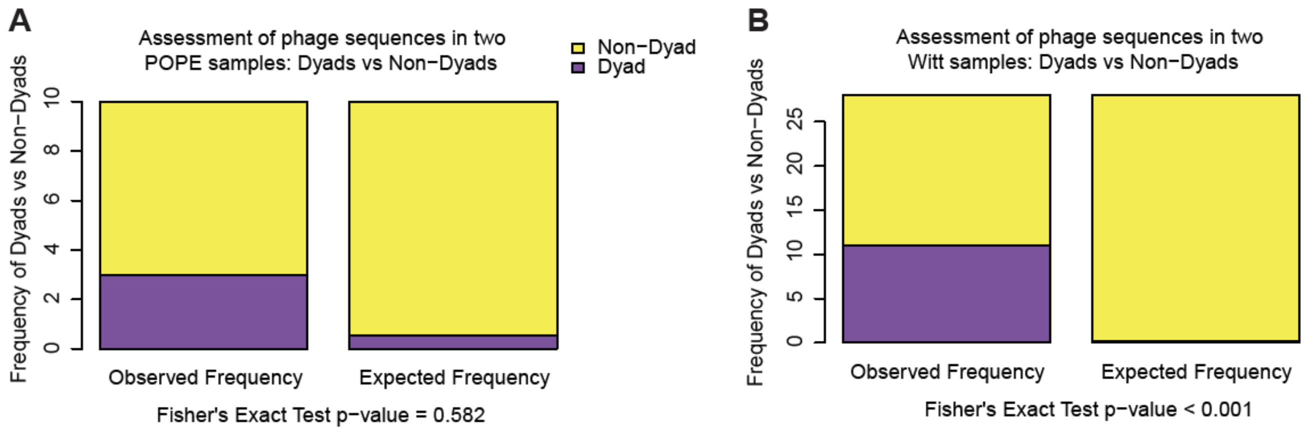

Supplemental Figure 1: Dyad frequency of phage sequences present in two samples. (**A**) The observed frequency of dyads (purple) among phage sequences identified in two POPE samples (left) compared to the frequency of dyads expected by random chance (right). (**B**) As in (**A**), using phage sequences identified in two samples from the Witt cohort. Non-dyads (yellow) are defined as any combination of two samples in the cohort other than the dyad combination, including maternal-maternal and infant-infant pairings.
